# Slope-Increasing Anterior Opening Wedge–Proximal Tibial Osteotomy for Posterior Knee Instability Due to Symptomatic Flat Posterior Tibial Slope

**DOI:** 10.1016/j.eats.2025.103766

**Published:** 2025-07-25

**Authors:** Riccardo Cristiani, Evan P. Shoemaker, Luke V. Tollefson, Dustin R. Lee, Matthew T. Rasmussen, Robert F. LaPrade

**Affiliations:** a Department of Molecular Medicine and Surgery, Stockholm Sports Trauma Research Center, Karolinska Institute, Stockholm, Sweden; b Capio Artro Clinic, FIFA Medical Centre of Excellence, Sophiahemmet Hospital, Stockholm, Sweden; c Twin Cities Orthopedics, Edina, Minnesota, U.S.A.

## Abstract

A flat posterior tibial slope increases posterior tibial translation and places additional stress on the posterior cruciate ligament. In cases where posterior cruciate ligament insufficiency is exacerbated by altered posterior tibial slope, such as following the malunion of a lateral tibial plateau fracture or anterior physeal arrest, a slope-increasing tibial osteotomy can be a valuable strategy to reduce posterior tibial translation and restore sagittal alignment. This Technical Note outlines the surgical approach for an anterior slope–increasing tibial osteotomy in the setting of chronic posterior knee instability associated with a flat posterior tibial slope following a malunion of a tibial plateau fracture.

Posterior tibial slope (PTS) is a critical determinant of knee biomechanics, especially in the setting of cruciate ligament function. Alteration of the PTS can result from native anatomy or prior trauma, such as a malunion following tibial plateau fracture or anterior physeal arrest. Although the native PTS is frequently reported to range from 7° to 9°, a decreased slope increases posterior tibial translation (PTT) and places additional stress on the posterior cruciate ligament (PCL).^1^^,^^2^ With concomitant PCL injuries, increased posterior knee laxity can be observed, which can be responsible for instability, pain, and degenerative joint changes over time.^3^ Conversely, intentionally increasing tibial slope transfers the added stress on the PCL to the anterior cruciate ligament, effectively offloading the PCL. Consequently, with this slope change, a decrease in PTT and tibial sag in knees with PCL injuries is often observed.^4^^,^^5^ A slope-increasing tibial osteotomy is an established technique that aims to restore sagittal alignment and reduce PTT, thus restoring native knee biomechanics.^2^^,^^6^, ^7^, ^8^ This Technical Note describes a slope-increasing anterior opening wedge–proximal tibial osteotomy (AOW-PTO) in the setting of chronic posterior knee instability resulting from a reduced tibial slope.

## Surgical Technique

A detailed demonstration of the patient evaluation, arthroscopic findings, and AOW-PTO is provided in Video 1. A step-by-step guide and surgical pearls are included in Table 1.

**Table 1:** Step-by-Step Surgical Guide and Key Pearls for Anterior Opening Wedge Proximal Tibial Osteotomy Posterior Knee Instability Due to Symptomatic Flat Posterior Tibial Slope

Step-by-Step Guide	Pearls
An anterior midline incision is made to fully expose the extensor mechanism and the osteotomy site.	The incision should extend from approximately 6 cm above the patella to 6 cm below the tibial tubercle.
Continue the dissection along both the medial and lateral borders of the patellar tendon.	To expose the margins of the opening wedge osteotomy, elevate the lateral periosteum and surrounding tissues over the anterior compartment. This dissection should extend from the lateral edge of the patellar tendon proximally toward the area anterior to the tibiofibular joint. On the medial side, elevate approximately 1 cm of periosteum, starting at the MCL and continuing completely beneath the patellar tendon.
Anteromedial and anterolateral parapatellar portals are created, followed by joint insufflation.	Evaluation of the menisci, cruciate ligaments, and the medial and lateral compartments is made. Any loose bodies or adhesions should be debrided within the suprapatellar pouch, medial and lateral gutters, and within the intra-articular compartment.
Attention is next directed to the slope-increasing AOW-PTO.	Two guide pins are inserted parallel to the tibial plateau, directed toward the posterior tibial cortex, one on each side of the patellar tendon, just above the tibial tubercle. As a general guideline, every 1 mm of wedge opening in a supratubercle opening wedge osteotomy results in approximately 1° of increased posterior tibial slope.
The AOW-PTO cuts are made.	A small ACL saw is used medially and laterally along the trajectory of the guide pins. The osteotomy is completed with an osteotome under fluoroscopic guidance, making sure that the posterior tibial cortex remains intact. A spreader device is positioned, and the osteotomy is gradually opened to the desired correction. The spreader is left in place for approximately 5 minutes to allow stress relaxation of the posterior cortical hinge.
The AOW-PTO is fixed and grafted.	Two Puddu plates are positioned, 1 medially and 1 laterally to the patellar tendon. Fixation is achieved with 4 screws on the medial plate and 2 screws on the lateral plate, which functions primarily as a buttress to maintain the osteotomy gap. Fluoroscopic images are obtained to verify that the intended correction has been successfully achieved. The osteotomy site is thoroughly packed with allograft bone graft.
The deep and superficial tissues are closed in layers.	A layered closure helps prevent the formation of a hematoma postoperatively.

ACL, anterior cruciate ligament; AOW-PTO, anterior opening wedge–proximal tibial osteotomy; MCL, medial collateral ligament; PCL, posterior cruciate ligament.

### Patient Evaluation

**Fig 1:**
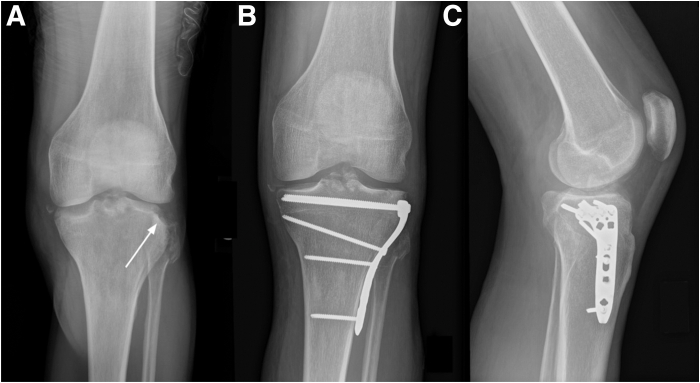
Left knee preoperative radiographs showing a lateral tibial plateau fracture: preoperative anteroposterior view (A) and postoperative anteroposterior (B) and lateral (C) views following open reduction and internal fixation.

**Fig 2:**
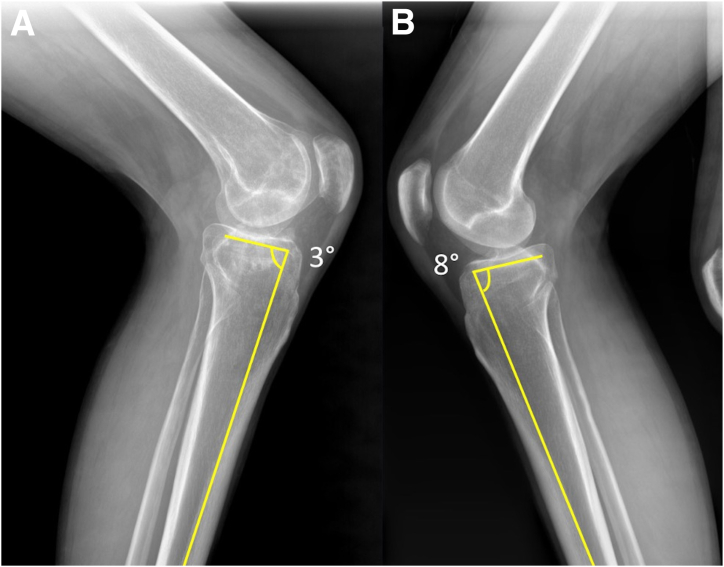
Preoperative lateral radiographs of the left knee following the removal of previously retained hardware. Bilateral standing lateral radiographs show a decreased posterior tibial slope of 3° on the left (A) compared to 8° on the right, uninjured knee (B).

**Fig 3:**
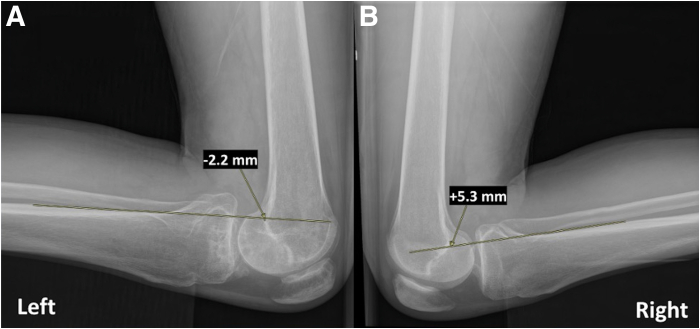
Kneeling posterior cruciate ligament (PCL) stress radiographs of the affected left knee (A) and the unaffected right knee (B) show a 7.5-mm side-to-side increased posterior tibial translation on the injured side. A line is drawn beginning 15 mm distal to the joint line along the posterior tibial cortex and continues proximally through the femoral condyles. A second perpendicular line is made from the posterior aspect of the Blumensaat line, and comparing this value to the normal contralateral knee is the amount of posterior tibial translation.

For patients with a history of persistent posterior knee instability, a thorough injury history (Fig 1), clinical examination, and radiographic (Figs 2, 3) assessment are essential for accurate evaluation and treatment planning. For patients with significantly decreased PTS and PTT instability, an AOW-PTO may be indicated.

### Anesthesia and Positioning

The patient is placed in the supine position and induced under general anesthesia. Knee range of motion and stability are carefully assessed to confirm preoperative clinical findings. A high-thigh tourniquet is placed on the surgical leg, which is then placed in an extremity holder (Mizho OSI), and the patient is administered perioperative cefazolin for prophylaxis against infection.

### Anterior Approach and Site Preparation

**Fig 4:**
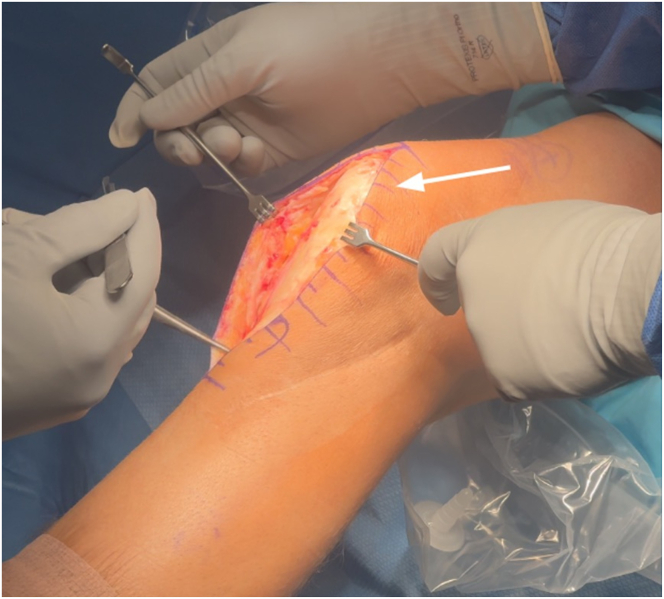
Left knee anterior midline incision for slope-increasing anterior opening wedge–proximal tibial osteotomy with the patient in the supine position. The incision is made approximately 6 cm proximal to the patella and extended 6 cm distal to the tibial tubercle, providing full exposure of the extensor mechanism (white arrow) and the planned osteotomy site. The margins of the patellar tendon are carefully dissected until completely exposed, and the paratenon is meticulously dissected and mobilized.

**Fig 5:**
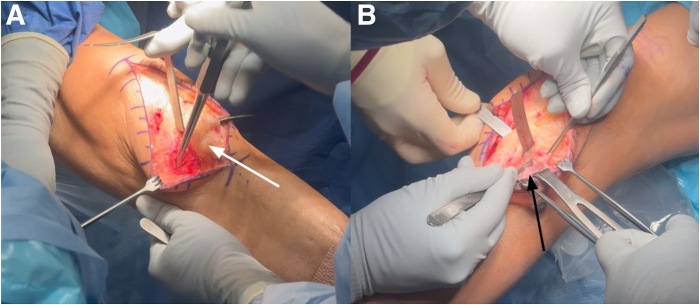
Left knee anterior midline incision with the patient in supine position. The exposure for the opening wedge osteotomy begins with elevation of soft tissue on both the medial and lateral sides of the patellar tendon. (A) Medially, a subperiosteal flap is developed (white arrow), beginning at the tibial tubercle and extending in a horizontal direction toward the medial collateral ligament, and elevated proximally by approximately 1 cm. (B) The lateral periosteum over the anterior compartment is elevated proximally for 1 cm (black arrow), extending from the lateral border of the patellar tendon to the anterior aspect of the proximal tibiofibular joint.

The open approach is performed first to allow for clear identification of anatomic structures before fluid extravasation from the arthroscopy. A central incision is made from the superior pole of the patella to the tibial tubercle. Careful dissection is carried out to expose the edges of the patellar tendon, and the paratenon is meticulously dissected and mobilized. The deep infrapatellar bursa is entered, and scar tissue is excised to improve visualization (Fig 4). A subperiosteal flap is then developed, beginning at the tibial tubercle and extending medially in a horizontal direction toward the medial collateral ligament, and elevated proximally by approximately 1 cm. A small Cobb elevator is inserted beneath the medial collateral ligament to facilitate the placement of a Z-retractor for soft tissue retraction. A similar dissection above the anterior compartment musculature is performed on the lateral side. Any scar tissue from the previous surgery is carefully excised, and a periosteal flap is elevated approximately 1 cm in length, starting from the anterior compartment and extending proximally to allow adequate exposure (Fig 5).

### Arthroscopy

Standard anteromedial and anterolateral arthroscopic portals are established. Debridement and lysis of adhesions is carried out beginning in the suprapatellar pouch and extending through the medial and lateral gutters into the anterior compartment. Any loose bodies within the intercondylar notch are removed, and an extensive notchplasty is performed using a burr to remove osteophytes and restore full knee extension. Arthroscopic evaluation of the cruciate ligaments, medial and lateral meniscus, and medial and lateral compartments is made.

### Slope-Increasing Anterior Opening Wedge–Proximal Tibial Osteotomy

**Fig 6:**
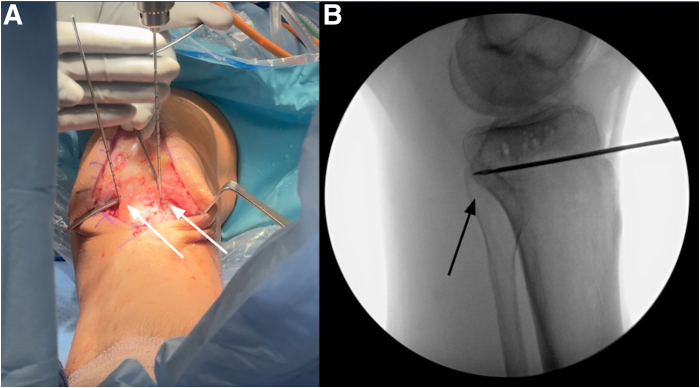
Left knee anterior midline approach with the patient in the supine position and lateral intraoperative fluoroscopic image. Two guide pins (white arrows) are inserted parallel to the tibial plateau toward the posterior cortex (black arrow), positioned on either side of the patellar tendon just above the tibial tubercle (A, B).

**Fig 7:**
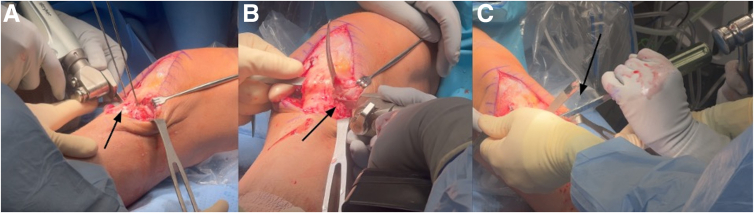
Left knee anterior midline approach with the patient in the supine position. A small oscillating anterior cruciate ligament saw blade is used to make cortical cuts along the guide pin trajectory medially (A) and laterally (B), and the osteotomy wedge is completed by carefully advancing an osteotome (C), preserving the posterior cortex and ensuring the cortical hinge remains intact to prevent any iatrogenic injury to the posterior neurovascular structures.

**Fig 8:**
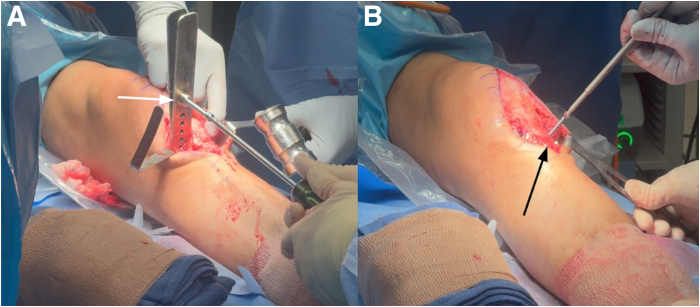
Left knee anterior midline approach with the patient in the supine position. A knee-opening spreader is positioned to gradually open the osteotomy to the desired correction and is held in place for approximately 5 minutes to allow posterior cortical hinge stress relaxation (A). A 7.5-mm posteriorly sloped Puddu plate (Arthrex) is applied medially, followed by placement of a second 7.5-mm plate laterally, adjacent to the patellar tendon, to prevent unintended closure of the lateral osteotomy gap. Fixation is achieved with 4 screws on the medial plate and 2 on the lateral plate (B), which functions primarily as a buttress.

Fluoroscopic imaging is utilized to assist with the osteotomy. Two guide pins are placed parallel to the tibial plateau, aimed toward the posterior cortex of the tibia, one on each side of the patellar tendon (Fig 6). A small oscillating saw is then used to make cortical cuts along the trajectory of the guide pins on both the medial and lateral sides. The osteotomy is carefully completed with an osteotome under fluoroscopic guidance, ensuring that the posterior tibial cortex remains intact (Fig 7). A knee-opening spreader device is positioned, and the osteotomy is gradually opened to the desired correction. The spreader is left in place for approximately 5 minutes to allow for stress relaxation of the posterior cortical hinge. A posteriorly sloped Puddu plate (Arthrex) is then applied medially. To prevent unintended closure of the lateral osteotomy gap, a second posteriorly sloped Puddu plate is placed laterally, adjacent to the patellar tendon. Fixation is achieved using 4 screws on the medial plate and 2 screws on the lateral plate, as this plate serves primarily as a buttress to maintain the osteotomy opening (Fig 8).

### Correction Evaluation and Bone Grafting

**Fig 9:**
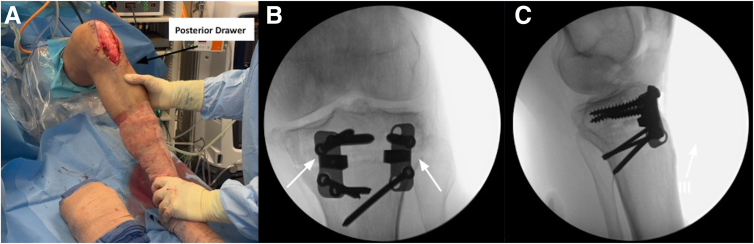
(A) Anterior view of the left knee during evaluation of posterior tibial translation with the posterior drawer test. Anteroposterior (B) and lateral (C) fluoroscopic images showing final positioning of medial and lateral plates (white arrows) and screws and bone grafting of the 7.5-mm wedge before final closure. For slope-increasing tibial osteotomies in the setting of decreased slope, restoration to the native posterior tibial slope ranges of 7° to 9° is desired or comparative alignment to the contralateral unaffected lower extremity.

A posterior drawer test is performed following the osteotomy and reveals only a trace amount of PTT. Anteroposterior and lateral fluoroscopic images are obtained (Fig 9). Confirmation of the desired PTS angle within the normal anatomic range is made. The osteotomy site is then thoroughly packed with an allograft bone graft.

### Final Closure

The tourniquet is released, and hemostasis is achieved. The deep tissues are closed using 0 and 2-0 Vicryl sutures, followed by Monocryl (Ethicon), for the skin closure. A sterile dressing and a knee immobilizer in full extension are applied.

### Postoperative Protocol

Physical therapy begins on postoperative day 1, with a particular emphasis placed on maintaining full knee extension. The patient has no restrictions on knee flexion. No weightbearing is recommended for the first 8 weeks postoperatively. At the 8-week mark, radiographs are obtained to assess osteotomy healing. If adequate healing is confirmed, the patient is allowed to begin a gradual return to weightbearing, increasing by 25% of body weight each week. At 12 weeks, repeat radiographs are performed to confirm continued healing, after which the patient may begin weaning off crutches. Follow-up imaging, including PCL stress radiographs, are be obtained at the 6-month mark to evaluate the effect of the increased tibial slope on the PCL and PTT.

## Discussion

The management of chronic posterior knee instability presents a significant clinical challenge. Decreases in PTS, which can result from previous trauma, such as malunion of tibial plateau fractures or anterior physeal arrest, can lead to increased PTT and genu recurvatum, as well as place greater mechanical stress on the PCL, thereby worsening instability.^1^^,^^2^ The current literature is limited primarily to case reports and small case series describing surgical techniques.^9^ Nonetheless, a slope-increasing osteotomy, although less frequently performed, has emerged as a valuable strategy in select cases of chronic PCL insufficiency, particularly in patients with altered sagittal alignment.^6^^,^^10^^,^^11^ By increasing the PTS, the osteotomy reduces PTT and excessive loading of the PCL, restoring more physiologic knee kinematics.^4^^,^^5^ The technique described in this Technical Note focuses on the approach for performing a slope-increasing osteotomy in a patient who has a flat PTS causing chronic posterior instability. This strategy not only improves sagittal alignment but also reduces posterior knee laxity, with the potential to reduce the need for PCL reconstruction. The advantages and disadvantages of this technique are presented in Table 2.

**Table 2:** Advantages and Disadvantages of Anterior Opening Wedge–Proximal Tibial Osteotomy Posterior Knee Instability Due to Symptomatic Flat Posterior Tibial Slope

Advantages	Disadvantages
Decreases posterior tibial translation	Invasive and technically demanding procedure
Decreases PCL loads	Potential complications such as under- or overcorrection, nonunion or delayed union, irritation from hardware
If concomitant ligamentous or meniscal injury is present, concurrent reconstruction can be performed with an AOW-PTO	Prolonged nonweightbearing period: nonweightbearing for 8 weeks, followed by a gradual increase of 25% body weight per week, reaching full weightbearing by week 12
Allows accurate, customized correction of the tibial slope, tailored to the extent of the initial deformity	Prolonged recovery

AOW-PTO, anterior opening wedge–proximal tibial osteotomy; PCL, posterior cruciate ligament.

## Disclosures

The authors declare the following financial interests/personal relationships which may be considered as potential competing interests: R.F.L. has received funding grants from Ossur, 10.13039/100009026Smith & Nephew, Arthroscopy Association of North America, and American Orthopaedic Society for Sports Medicine; is a consultant or advisor for Ossur, Smith & Nephew, and Responsive Arthroscopy; has received travel reimbursement from Smith & Nephew; has received speaking and lecture fees from Foundation Medical, LLC; and has a patent with royalties paid to Ossur. All other authors (R.C., E.P.S., L.V.T., D.R.L., M.T.R.) declare that they have no known competing financial interests or personal relationships that could have appeared to influence the work reported in this paper.

## Supplementary Data

Video 1 This is a video technique describing a slope-increasing anterior opening wedge–proximal tibial osteotomy. The patient is positioned supine, and a thorough examination under anesthesia is conducted to confirm the preoperative clinical findings. An anterior midline incision is made, extending from 6 cm proximal to the superior pole of the patella to 6 cm distal to the tibial tubercle. Dissection is carried laterally to the region anterior to the tibiofibular joint and medially along the medial joint line to adequately expose the osteotomy site. Standard anteromedial and anterolateral parapatellar portals are created. Evaluation of the cruciate ligaments, menisci, and medial and lateral compartments is made. A lysis of adhesions is performed in all compartments, loose bodies within the intercondylar notch are removed, and an extensive notchplasty is carried out to remove osteophytes and restore full knee extension. Intra-articular injuries are verified. Two guide pins are inserted parallel to the tibial plateau, directed toward the posterior tibial cortex, one on each side of the patellar tendon, just above the tibial tubercle. A small anterior cruciate ligament saw is used medially and laterally along the trajectory of the pins. An osteotome is used to complete the osteotomy, keeping the posterior tibial cortex intact. A spreader device is positioned, and the osteotomy is gradually opened to the desired correction. Two 7.5-mm posteriorly sloped Puddu plates are positioned, 1 medially and 1 laterally to the patellar tendon. Fixation is achieved with 4 screws on the medial plate and 2 screws on the lateral plate, which functions primarily as a buttress to maintain the osteotomy gap. The osteotomy site is thoroughly packed with allograft bone graft. The deep and superficial tissues are closed with a suture.
